# Characterizing withdrawal from long-acting injectable buprenorphine: An observational case series

**DOI:** 10.1016/j.dadr.2025.100329

**Published:** 2025-04-05

**Authors:** Victoria Hayes, Llewellyn Mills, Gaye Byron, Carolyn Stubley, Eleanor Black, Benjamin T. Trevitt, Andrew A. Somogyi, Arshman Sahid, Nicholas Lintzeris

**Affiliations:** a Drug and Alcohol Services, South Eastern Sydney Local Health District, New South Wales, Australia; b Specialty of Addiction Medicine, Faculty of Medicine and Public Health, University of Sydney, New South Wales, Australia; c Drug and Alcohol Clinical Research and Improvement Network (DACRIN), Sydney, New South Wales, Australia; d We Help Our Selves (WHOS), Lilyfield, Sydney, New South Wales, Australia; e Alcohol and Drug Services, Metro North Royal Brisbane Hospital, Brisbane, Queensland, Australia; f Discipline of Pharmacology, Faculty of Health and Medical Science, University of Adelaide, Adelaide, South Australia, Australia

**Keywords:** Opioid withdrawal, Buprenorphine, Long-acting injectable buprenorphine, Opioid dependence treatment

## Abstract

**Introduction:**

Long-acting injectable buprenorphine (LAIB) products are being increasingly used to treat patients with opioid dependence. Limited data is available on the severity or timespan (time to onset, peak, duration) of withdrawal signs and symptoms following discontinuation of treatment.

**Methods:**

Participants aiming to discontinue long-term LAIB treatment commenced the study on the day of their final dose of Buvidal® 64 mg Monthly. Participants were monitored with weekly assessments of withdrawal severity, cravings, general health, and patient experience measures for up to 16 weeks after last dose.

**Results:**

Fifteen participants – those who remained for at least four weeks after the last LAIB dose – were included in the study. There was minimal increase in withdrawal severity over the study period, with an average peak Clinical Opioid Withdrawal Scale score of 4.8 ± 2.7, occurring at a median of 6 weeks (IQR 4–7.5) after the last LAIB dose. Cravings scores were generally low but increased gradually over the 16-week study period. There was no deterioration in physical or mental health scores, and participants reported high levels of satisfaction with the withdrawal experience. Ten participants used rescue medications, predominately in weeks 5 or 6 after the last dose.

**Discussion and conclusions:**

Participants (last dose of Buvidal® 64 mg Monthly) experienced minimal or mild withdrawal signs and symptoms, usually peaking in severity between 5 and 8 weeks after the last dose. These results are encouraging, however clinical trials comparing withdrawal outcomes between LAIB, sublingual buprenorphine (SL BPN) and methadone are required to inform treatment planning.

## Highlights

• Long-acting injectable buprenorphine (LAIB) is widely used, yet little is known about the experience of discontinuing.
• We tracked the experiences of 15 people discontinuing LAIB for up to 16 weeks in a residential rehabilitation unit.
• Stopping LAIB does not appear to be associated with severe withdrawal or cravings, but larger-scale trials are necessary.
• Studies comparing LAIB withdrawal to sublingual buprenorphine or methadone are needed to better inform treatment planning.

## 1 Introduction

Long-acting injectable buprenorphine (LAIB) products are being increasingly used to treat opioid use disorder, usually in the form of monthly subcutaneous injections. Whilst much is known about the pharmacokinetics, efficacy and safety of LAIB treatment (Albayaty et al., 2017, Haight et al., 2019, Jones et al., 2021, Lintzeris et al., 2024, Lofwall et al., 2018, Marsden et al., 2023, Rosenthal et al., 2016, Sigmon et al., 2006), questions remain regarding the onset, duration and severity of opioid withdrawal syndrome following discontinuation of LAIB treatment.

Successfully ceasing opioid agonist treatment (OAT) is identified as a key goal by many patients (Clay et al., 2023, Lenné et al., 2001, Stancliff et al., 2002, Winstock et al., 2011). However, cessation of methadone or sublingual buprenorphine (SL BPN) is often associated with prolonged and/or severe opioid withdrawal syndrome with high rates (greater than 85 %) of return to regular opioid use (Lenné et al., 2001, Winstock et al., 2011). The standard approach in many clinical settings is to gradually taper doses over weeks to months, with withdrawal discomfort typically most severe in the several days or weeks after the final opioid dose (Reed et al., 2007, Seifert et al., 2002). Despite early enthusiasm for the use of SL BPN to assist methadone patients to successfully cease treatment (Breen et al., 2003, Winstock et al., 2009), systematic reviews suggest no obvious advantages in using SL BPN over methadone for withdrawal from OAT (Gowing et al., 2017).

There is considerable hope that LAIB formulations may prove to be a satisfactory and effective approach for ceasing OAT. The pharmacokinetic profile of Buvidal Monthly (terminal plasma half-life of 19–25 days), results in a delayed and gradual reduction in plasma concentrations over weeks after the last dose (Björnsson et al., 2023, Jones et al., 2021, Sigmon et al., 2006), which should theoretically be associated with mild opioid withdrawal symptoms. One small study (Sobel et al., 2004) examined withdrawal outcomes in five opioid-dependent participants following a single 58 mg dose of a different depot subcutaneous formulation (Norvex®), delivering gradually tapering buprenorphine plasma concentrations over four to six weeks. The study reported minimal subjective or objective withdrawal features in the six weeks after dosing with no supplementary medications required. The small subject numbers, limited follow-up interval (six weeks) after a single LAIB dose, and the use of an LAIB formulation that is not commercially available restricts the conclusions we can make from this study.

More recently, Ritvo and colleagues (2021) reported on three cases who were prescribed low-dose SL BPN for opioid use disorder (OUD). They were transitioned to a single dose of 100 mg LAIB (Sublocade®) which was ceased without eliciting any opioid withdrawal symptoms. Rodriguez and Suzuki (2023) documented a further four cases with OUD who were treated with LAIB (Sublocade) for varying durations (4–11 months) which they ceased with minimal-to-no opioid withdrawal symptoms. However, both case series were conducted in outpatient settings without confirmation of abstinence from other opioids or other drugs (e.g. urine tests), nor structured assessment of withdrawal severity using validated measures.

Nevertheless, these published case reports, together with positive anecdotal reports (of minimal withdrawal discomfort) from patients in our own clinical practice, has led us to hypothesize that patients can discontinue LAIB treatment without severe withdrawal discomfort.

The primary objective of this study was to describe the profile of withdrawal signs and symptoms in participants discontinuing opioid treatment with Buvidal Monthly in a long-term residential treatment setting. Specifically, we aim to describe the onset, time to peak severity and duration of opioid withdrawal features over an extended inpatient admission, and to examine related outcomes such as cravings for opioid use, general health outcomes and other experiences of people who have discontinued LAIB.

## 2 Methods

### 2.1 Study design and setting

The study was an open-label prospective case series examining withdrawal following abrupt discontinuation of LAIB Monthly treatment. An inpatient residential setting was considered necessary to examine withdrawal experiences, away from the risks of unsanctioned opioid or other substance use in the community - which could interfere with interpretation of withdrawal outcomes. The study was conducted at We Help Ourselves (WHOs; https://www.whos.com.au), a rehabilitation program for patients seeking to withdraw from OAT medications in a supported residential environment. It routinely offers a three to six-month treatment episode, during which opioid medications (methadone or SL buprenorphine) are tapered over the first four to 12 weeks of treatment, individualized for each patient.

### 2.2 Participants

Participants were recruited through routine intake at the residential treatment service. To be included in the study participants needed to: (a) be 18 or over; (b) have been in continuous OAT for at least six months prior to admission, with at least eight weeks of continuous buprenorphine treatment; (c) have a desire to cease BPN treatment; (d) not be withdrawing from other substances at admission; (e) have no severe medical or social conditions (e.g. upcoming custodial sentence) which might impact their capacity to withdraw from opioids or remain in the study; (f) have had no significant changes in other psychoactive medications (antidepressants, antipsychotics or anticonvulsants) in the four weeks prior to admission; (g) having received treatment with Buvidal monthly (64 mg, 96 mg or 128 mg) for at least one dose prior to study admission; (h) remaining enrolled in the study for more than four weeks (i.e. ≥29 days) after their final dose of buprenorphine. The requirement of remaining in the unit for at least four weeks after the last Buvidal Monthly dose reflects the routine dosing schedule for patients with Buvidal Monthly (doses administered every four weeks) – whereby patients are not considered to have ‘discontinued’ Buvidal until reaching the 4-week time point.

We aimed to recruit 15 participants who met the above eligibility criteria

We note that the original study design was a between-subjects non-randomised case-series comparing two groups: those discontinuing Buvidal and a group discontinuing sublingual buprenorphine by tapering doses to 0 mg (see Australia New Zealand Clinical Trial Registry # ACTRN12621001011875). We aimed to recruit fifteen eligible participants to each arm; however, only two participants chose to taper off sublingual buprenorphine during the twenty-month recruitment period (03/09/2021 – 27/07/2023) after which the study was discontinued due to timeline/budgetary constraints. Two participants are insufficient for meaningful between-group comparisons; therefore we focus on describing the Buvidal group in the main manuscript, and present the two sublingual buprenorphine participants’ data in supplementary materials.

### 2.3 Intervention

Participants received a single dose of Buvidal Monthly 64 mg SC injection on day 1 of the study, with no further BPN provided.

Limited “rescue” medications – temazepam for insomnia, metoclopramide for nausea/vomiting, hyoscine butylromide for abdominal cramps, and Lomotil® [diphenoxylate +  atropine sulphate] for diarrhoea – were available on request for participants who were unable to tolerate withdrawal discomfort during their admission (see Supplementary Materials for details, eTable 1).

**Table 1:** Baseline characteristics (*N* = 15).

**Age**, yrs-old, M (SD)	37.1 (5.2)
**Gender**, n (%)	
Male	11 (73 %)
Female	4 (27 %)
**Aboriginal**, n (%)	3 (20 %)
**Education**, n (%)	
Left before Yr 10	4 (27 %)
Attained Yr 10	5 (33 %)
Attained Yr 12	2 (13 %)
Attained Tertiary	4 (27 %)
**Living situation**, n (%)	
Renting	6 (40 %)
Own house/mortgage	1 (7 %)
Lives with family/friends	4 (27 %)
Homeless	4 (27 %)
**Relationship status**, n (%)	
Single/Separated	11 (73 %)
Married/Defacto	4 (27 %)
**Has children**, n (%)	6 (40 %)
**Current legal issues**, n (%)	8 (53 %)
**Any substance use in previous 28 days**, n (%)	
Alcohol	1 (7 %)
Cannabis	0 (0 %)
Amphetamine	3 (20 %)
Benzodiazepine	4 (27 %)
Heroin	2 (13 %)
**Opioid use history**	
Age of first regular use, yrs-old, M (SD)	21.7 (6.8)
Age of first opioid treatment, yrs-old, M (SD)	26.4 (7.4)
Heroin main type of opioid, n (%)	14 (93 %)
Came off OAT for 1 month or more once or more, n (%)	12 (80 %)
When came off OAT (*n* = 12), stayed off for ≥ 6 months, n (%)	6 (50 %)
**Depression Anxiety Stress Scale**, n (%)	
Depression	
Normal	8 (53 %)
Mild	1 (7 %)
Moderate	3 (20 %)
Severe/Extremely Severe	3 (20 %)
Anxiety	
Normal	6 (40 %)
Mild	5 (33 %)
Moderate	3 (20 %)
Severe/Extremely Severe	1 (7 %)
Stress	
Normal	7 (47 %)
Mild	3 (20 %)
Moderate	4 (27 %)
Severe/Extremely Severe	1 (7 %)
**Detoxification Fear Survey Schedule^a^** – 14, n (%)	
Problematic fear of withdrawal	5 (33 %)
No problematic fear of withdrawal	10 (67 %)
**Treatment Satisfaction Questionnaire – Medication**, M (SD)	
Convenience	79.1 (15.4)
Effectiveness	75.9 (13.9)
Side effects	88.3 (17.3)
Global satisfaction	75.2 (21.2)

Participants engaged in routine group activities within the residential unit, with access to peer support, nursing, medical and psychosocial services available as part of routine care.

### 2.4 Outcomes and measures

Opiate withdrawal severity as measured by the *Clinical Opioid Withdrawal Scale* (COWS; Wesson and Ling, 2003; weekly) was the primary outcome for the study. The COWS is an 11-item scale capturing objective and subjective dimensions of the withdrawal syndrome. Total scores range from 0 (no withdrawal) to 48 (Severe withdrawal), with mild withdrawal scoring 5–12, moderate withdrawal 12–24, and severe withdrawal > 25.

Secondary outcome measures included:

• *Subjective Opioid Withdrawal Scale* (SOWS; Handelsman et al., 1987; weekly): 16 items self-rated by participants measuring subjective withdrawal symptoms. Scores range from 0 to 64 with mild withdrawal scores of 1–10, moderate withdrawal 11–20, and severe > 20.
• *Objective Opiate withdrawal Scales* (OOWS; Handelsman et al., 1987; weekly): This 13-item scale (scores 0–13), completed by trained clinicians (e.g. nurse, medical officer), assesses objective signs of opioid withdrawal.
• *Opioid Craving Scale* (OCS; McHugh et al., 2014; weekly): This three-item scale measures opioid cravings on a 0 (no craving) to 10 (severe craving) response scale for each item: (i) at the time the respondent is completing the questionnaire, (ii) over the previous week when the respondent is reminded of opioids, and (iii) when the respondent imagines using opioids in their usual environment.
• *Treatment Satisfaction Questionnaire – Medication* (TSQM; Atkinson et al., 2004; 4-weekly). The TSQM measures respondents’ satisfaction with their medication, with four subscales: Convenience, Side effects, Effectiveness, and Global satisfaction. Scores for each factor range from 0 to 100 with higher scores representing greater satisfaction, with a threshold score of 80 being considered high level of satisfaction in patients with chronic diseases (Radawski et al., 2019). As participants in this study did not take LAIB past Day 1, items designed to measure convenience of medication administration were omitted. Structured questions were also administered at the exit research interview regarding how participants rated the current quit attempt compared with prior attempts, and reasons for discontinuing the study.
• *Patient Reported Outcome Measurement Information System* (PROMIS-29; Cella et al., 2010; 4-weekly): this validated questionnaire assesses general health status and quality of life, with subscales of social function, physical function, anxiety, depression, fatigue, sleep disturbance and pain interference. Subscales are expressed as *t*-scores, with scores normed to the overall population with a mean of 50 and a standard deviation of 10.
• *Depression Anxiety and Stress Scale* (DASS; Lovibond and Lovibond, 1995; Day 1 and study end): measuring severity of respondents’ depression, anxiety, and stress, with 21 items grouped into three 7-item factors (response scale 0–3), each yielding a composite score out of 21, with higher scores indicating more severe problems.
• Treatment completion rates and use of rescue medications were extracted from medical records. Adverse events were assessed by study medical officers.
• Urine drug screens collected at 4-weekly interviews (4, 8, 12, 16) and blood samples collected at Week 12 for assessment of buprenorphine and norbuprenorphine concentrations, were quantified by LC-MS/MS (Musolino et al., 2022). Urine samples had a 1/5 dilution factor with a lower limit of quantification of both analytes of 0.5 ng/ml; for plasma, the lower limit of quantification was also 0.5 ng/ml.
• Participants’ previous history with opioid use (age first regular use, prior quit attempts) was assessed at enrolment. The Detoxification Fear Survey Schedule (DFSS; Milby et al., 1987) was administered at enrolment – a 14-item scale designed to measure respondents’ anxiety surrounding withdrawal from opioid treatment, with scores > 33 indicative of problematic fear of detoxification.
• Objective (actigraphy) and subjective (daily 1-min questionnaires) sleep measures were included in the study but require more detailed reporting than is possible within the limits of this paper and will be reported in a subsequent journal paper.

The study was approved by the South Eastern Sydney Local Health District Human Research Ethics Committee (Approval # 2020/ETH01924) and is registered with the Australia New Zealand Clinical Trial Registry (# ACTRN12621001011875).

### 2.5 Statistical analysis

We used Bayesian parameter estimation for the analyses. Bayesian parameter estimation provides similar estimates to equivalent regression models using standard Null Hypothesis Significance Testing (i.e. point estimates and 95 % intervals) but does not require correction for multiple comparisons (thereby reducing incidence of Type-2 error) and its results are not compromised by changes in recruitment goals (i.e. the study’s ‘stopping rule’) (Kruschke, 2014). Rate of change in each outcome over the (up to) sixteen-week trial was estimated via a mixed-effects model, with time (linear term, in weeks, range 1–16) as the fixed factor and participant ID as the random factor. Linear change is rare in natural systems therefore we included a quadratic term (i.e. time squared) as an additional fixed factor in these models to estimate whether rate of change in the outcome increased or decreased over time. Parameters were estimated via four chains of 10,000 estimates each. Flat or broad weakly-regularising priors were used for all intercepts, betas, and variance parameters (see online supplementary file for details). Diagnostics – $\overset{®}{\text{R}}$, effective sample size and trace plots – were performed to assess convergence. As the word ‘significant’ has a specific, frequentist meaning we use the words ‘notable’ or ‘noteworthy’ instead. We considered any estimate whose 95 % Credible Interval (CI) excluded 0 to be noteworthy.

Maximum withdrawal scores and time to maximum score (in weeks) were calculated for each participant who stayed beyond four weeks.

All analyses were performed in R (R Core Team, 2016), version 4.2.2, using the tidyverse (Wickham, 2017), brms (Bürkner, 2017) and emmeans (Lenth, 2020) packages. Data and code used for this manuscript is publicly available at https://osf.io/7zs2q/.

## 3 Results

### 3.1 Recruitment and study retention

**Fig. 1:**
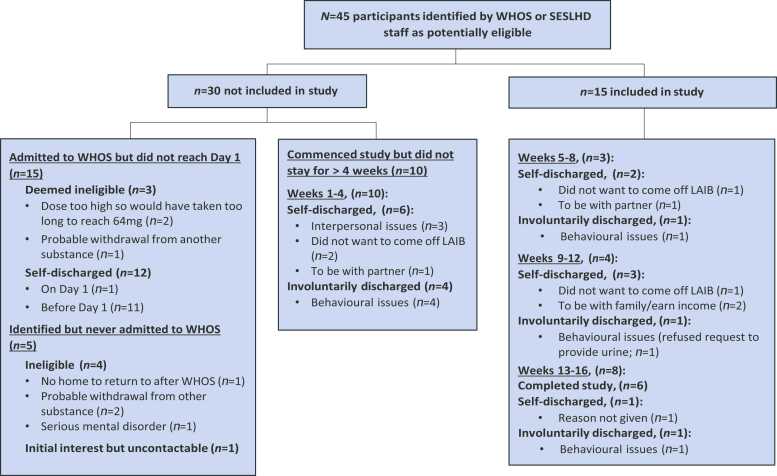
Study Flow Diagram.

Details of recruitment are outlined in Fig. 1. Forty-five patients were identified as potentially eligible, of whom 30 were not included. Of this 30, 20 were either deemed ineligible and never admitted to WHOS (*n* = 5) or admitted to WHOS but did not reach Day 1 of the study (*n* = 15). 10 commenced the study but did not stay in the study beyond 4 weeks after their last dose of Buvidal: One self-discharged unexpectedly to be with their partner who had been released from jail, three self-discharged due to interpersonal issues with fellow residents and/or finding the residential community setting unpleasant, two self-discharged at 26 and 28 days (when they would have been due their next dose had they not enrolled in the study) because they wanted to stay on Buvidal, and four were discharged involuntarily from WHOS for behaviours that violate their code of conduct (e.g. threatening other residents).

Fifteen participants stayed in the study beyond week 4 as per the planned recruitment strategy. Six of the 15 completed all 16 weeks, with nine leaving before 16 weeks. Two of these nine participants decided they did not want to discontinue LAIB treatment due to risks of resuming opioid use on return to the community, three indicated that they felt that they had largely completed withdrawal and were keen to return to their regular lives (e.g. work), three were discharged for behavioural reasons, and one self-discharged without providing a reason (see Fig. 1). The median duration of stay for the 15 participants was 71 days (range 40–112, Panel (a), Fig. 3).

### 3.2 Sample characteristics

Participants’ characteristics are reported in Table 1. Average age was 37.1 ± 5.2 years, most (73 %; 11/15) were male, and 20 % (3/15) identified as Aboriginal. Average age of participants’ first regular use of opioids was 21.7 ± 6.8. Most (80 %, 12/15) had previously withdrawn from OAT and stayed opioid-free for one month or more. A third (33 %, 5/15) had DFSS scores indicating a problematic fear of withdrawal (Milby et al., 1987). Participants indicated generally high levels of satisfaction with Buvidal at enrolment, with mean scores above 75/100 on all four subscales of the TSQM. Characteristics of the 10 participants who left before Day 29 and the two participants who chose to withdraw from Buvidal are reported in eTable 3 of the Supplementary Materials.

### 3.3 Withdrawal and craving

**Fig. 2:**
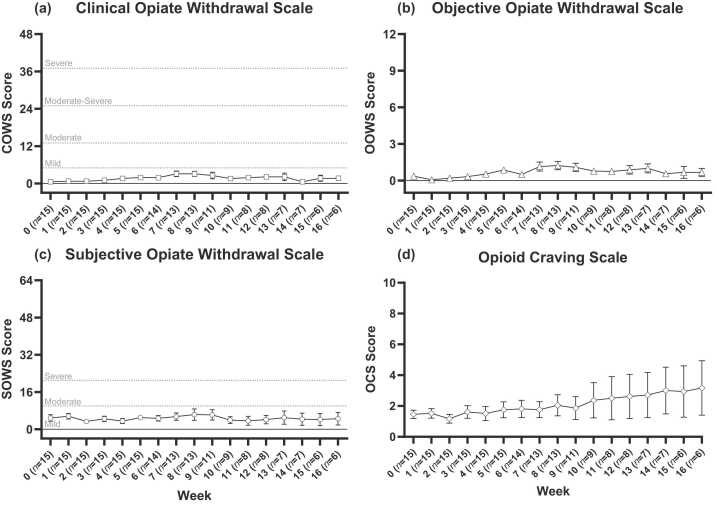
Withdrawal and craving across the 16-week trial.

Average withdrawal and cravings scores are presented in Fig. 2, whilst scores for individual participants are shown in Supplementary Materials, eFigures 1 and 2. Average weekly COWS scores remained below the threshold for mild withdrawal of 5 at all time points. Maximum COWS score remained below the mild withdrawal threshold for 10 participants (67 %) and was mild for the remaining five (33 %), and average maximum COWS score was also below this threshold (4.8 ± 2.7). There was a small but noteworthy per-week linear increase in COWS scores of 0.51 (CI: 0.32, 0.71) across the 16 weeks, accompanied by a noteworthy negative quadratic trend, with the linear per-week increase reducing by 0.03 points per week on average (estimate=−0.03; CI: −0.04, −0.02; see Table 2), implying that withdrawal increased over time but that this increase itself reduced over time.

**Table 2:** Average maximum score, average time to maximum score, and estimated average per-week change in withdrawal and craving (*N* = 15).

		**tmax**^b^		**Average change per week**	
**Outcome**	**Max**^a^ M (SD)			**Linear** Estimate (95 %CI)	**Quadratic** Estimate (95 %CI)
		M (SD)	Median (IQR)		
**Clinical Opioid Withdrawal Scale**, range 0–48	4.8 (2.7)	6.2 (3.0)	6 (4, 7.5)	**0.51 (0.32, 0.71)**	**−0.03 (−0.04, −0.02)**
**Objective Opioid Withdrawal Scale**, range 0–13	2.0 (1.1)	4.9 (2.6)	5 (3, 7)	**0.19 (0.10, 0.28)**	**−0.01 (−0.02, −0.00**^a^**)**
**Subjective Opioid Withdrawal Scale**, range 0–64	11.2 (7.8)	4.3 (3.6)	4 (1, 6.5)	0.13 (−0.42, 0.68)	−0.01 (−0.03, 0.02)
**Opioid Craving Scale**, range 0–10	3.4 (2.9)	5.3 (4.6)	5 (1, 8)	0.09 (−0.13, 0.30)	0.00 (−0.00^b^, 0.01)

a Estimate rounded up to −0.00, actually −0.004.

b -0.004

Average weekly scores on the SOWS remained within the 1–10 range (out of a possible 64), considered to be mild withdrawal (there is no “no withdrawal” score for the SOWS) (Fischer et al., 2021). Eight participants (53 %) met criteria for mild withdrawal, six (40 %) for moderate withdrawal, and one (7 %) for severe withdrawal. Average maximum SOWS scores were just above this threshold for mild withdrawal (11.2 ± 7.8). There were no noteworthy linear or quadratic trends in SOWS scores. Average OOWS scores also remained low (below 1.5 out of a maximum 13, and average maximum score of 2.0 ± 1.1). Similar to the COWS scores, there were noteworthy positive linear (estimate=0.19; CI: 0.10, 0.28) and negative quadratic (estimate=−0.03; CI: −0.04, −0.02) trends in average OOWS scores across the weeks, however once again these were too small to be considered clinically meaningful. Peak COWS, SOWS and OOWS occurred at a median of 5 or 6 weeks after the last Buvidal dose.

Average scores on the Opioid Craving Scale were also low for the first 6 weeks (<2 out of a possible 10). Although average scores appeared to increase towards the end of the trial there were no notable linear or quadratic trends.

While average scores remained low, individual scores varied across participants and timepoints (as shown in eFigures 1–3). The two participants who chose to withdraw from Sublingual buprenorphine had scores on the COWS, SOWS, and OOWS that remained low for the first 6–8 weeks whilst they were receiving SL buprenorphine doses of 4 mg or more, but these scores increased in both participants after they reduced to 2 mg SL BPN doses shortly before they left the study – one self-discharged, the other discharged involuntarily for testing positive for amphetamines in one of the compulsory urine tests WHOS administers to its clients (and hence deemed an administrative discharge). Cravings increased steadily for one of these participants but remained low for the other. It should be noted that two participants is insufficient data to make any generalisations or comparisons between groups.

### 3.4 Participant experience

**Fig. 3:**
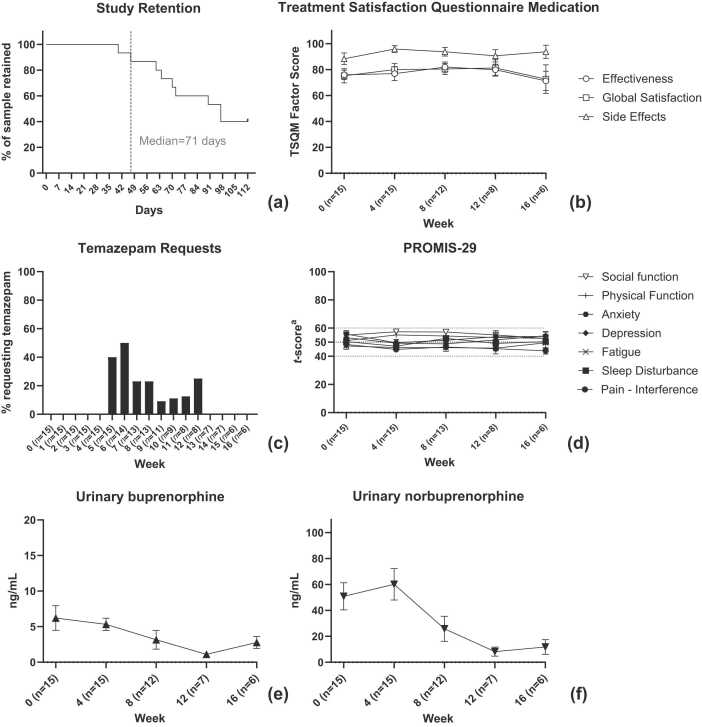
Other outcomes across the 16-week trial **a:***t-score normed to population mean = 50, SD = 10*.

TSQM satisfaction scores are shown in Fig. 3 (panel b). Ratings of Buvidal’s effectiveness, its side-effects and overall satisfaction remained high throughout the trial (range: 71.3–95.8). None of the three factors had noteworthy rates of change over time and there were no noteworthy pairwise differences in rates of change between any of the three factors.

Ten participants completed exit interviews with researchers regarding their experience of withdrawal compared to previous attempts. All ten indicated that this attempt to quit was better than their previous attempt to discontinue OAT, six indicating “Very much better”, three “Much better”, and one “A little better”.

### 3.5 General health measures

PROMIS-29 subscale scores are shown in Fig. 2 (panel d). Average scores for all subscales and all timepoints remained within one standard deviation (i.e. 10) of the normal population (i.e. 50). None of the seven factors had noteworthy rates of change over time and there were no noteworthy pairwise differences in rates of change between any of the seven factors.

Ten respondents completed DASS-21 questionnaires at research exit interviews. There were average reductions in scores between study entry and exit for all three factors (Depression 2.2 ± 2.7; Anxiety 2.0 ± 3.4; Stress 2.6 ± 3.8) but only for only Depression (estimate=2.1, CI: 0.2, 3.9) and Stress (estimate=2.5, CI: 0.1, 4.9) were these reductions noteworthy.

### 3.6 Use of rescue medications and adverse events

The most common rescue medication requested was temazepam. Ten of the 15 participants (67 %) took at least one 10 mg dose of temazepam during their stay: two had one dose, two had three doses and seven had all five possible doses. Temazepam was most commonly administered during Week 6 (see Fig. 2, panel c). Metoclopramide (10 mg oral) was administered nine times in total across two participants, in both cases during weeks 4 or 5. Hyoscine butylromide (for abdominal cramps) and Lomotil (for diarrhoea) were administered once each to the same lone participant, on days 44 and 46 respectively.

Eight of the 15 participants experienced one or more adverse events. There were 33 adverse events in total during the trial across these eight participants (whole sample: mean=2.2 ± 3.3, Median [IQR] = 1 [0, 2.5]; excluding the seven who experienced no events: mean=4.1 ± 3.6; Median [IQR] = 2.5 [1.75, 6]). All adverse events were rated as mild, and none were assessed as being related to study medication.

### 3.7 Urine and plasma levels of buprenorphine

Urinary buprenorphine and norbuprenorphine concentrations are reported in Fig. 2, panels e and f. There were notable linear reductions in both buprenorphine (estimate=-0.30 ng/ml per week, CI: −0.54, −0.07) and norbuprenorphine (estimate=-3.3 ng/ml per week, CI: −5.2, −1.4) across the trial period but no noteworthy quadratic trends.

Five participants had successful Week-12 blood draws. Buprenorphine and norbuprenorphine concentrations for two of these five were as follows: 2.1 (buprenorphine) & 5.1 ng/ml (norbuprenorphine) and 0.7 (buprenorphine) & 19.3 ng/ml (norbuprenorphine). Concentrations for the three remaining were below the lower limit of quantification of 0.5 ng/ml.

## 4 Discussion

The participants in our study discontinuing Buvidal Monthly 64 mg experienced minimal withdrawal severity, with peak signs and symptoms occurring between weeks five and eight (median six weeks) after the last injection. The increase in global withdrawal (COWS), subjective (SOWS) and objective (OOWS) features of withdrawal were minimal (e.g. an average increase in COWS scores of 0.5 [out of 48] from baseline), and unlikely to be clinically meaningful. Global patient perspectives also indicate that participants rated withdrawal discomfort as minimal, with most rating the withdrawal process as “very much” or “much better” than prior attempts at discontinuing sublingual buprenorphine treatment. Requests for temazepam for insomnia were also greatest in weeks 6–8 with two thirds of participants (10/15) requesting at least one dose. Overall the results indicate mild, tolerable withdrawal symptoms peaking between 5 and 8 weeks but reducing beyond this window.

Despite rapid adoption of long-acting injectable buprenorphine as a first line treatment for opioid use disorder in some countries (Lintzeris et al., 2024), only a handful of studies (e.g. Ritvo et al., 2021; Rodriguez and Suzuki, 2023; Sobel et al., 2004) have attempted to systematically describe people’s experience during withdrawal. Our study is the largest study to date to do so and is the first to describe withdrawal for patients using validated withdrawal scales and in a residential treatment environment, without the potential confounds of heroin or other substance use during the withdrawal period, and with minimal cues for return to opioid use compared to some patients' usual home environment. In one sense this is a strength of the study as it enhances our confidence regarding the characterisation of the LAIB withdrawal syndrome. However, in another sense the residential treatment setting is a limitation, since these findings may not generalise to patients discontinuing LAIB in the community – where drug cues and social conditions may impact upon subjective withdrawal severity, cravings and resumption of opioid or other substance use. Clinical trials in community settings are required to describe ‘naturalistic’ clinical outcomes (e.g. completion rates, substance use, withdrawal severity, cravings) of withdrawal from LAIB. Nevertheless, these findings enable us to provide better information for patients and clinicians regarding the likely profile (severity, time frame) of the withdrawal from monthly LAIB.

Our study did not contain a comparison group. An important next step therefore is to compare withdrawal from depot buprenorphine to withdrawal from sublingual in a randomised clinical trial. Only then will we be able to make more confident assertions about the severity of withdrawal from depot buprenorphine compared to sublingual. Nevertheless, our findings in this group of patients suggests withdrawal to be less severe than previously described in studies of patients discontinuing sublingual buprenorphine (Comer et al., 2020, Dakwar and Kleber, 2015) or oral methadone (Buydens-Branchey et al., 2005, Liu et al., 2013) treatment.

Another study limitation is the high proportion of LAIB participants (9/15, 60 %) who left the study after week 4 and before week 16. As such, we do not have complete data (to week 16) for these participants, and hence cannot be certain that they would not experience withdrawal following their study discontinuation. In addition, 50 % of participants in our study had made one or more past attempts to discontinue OAT, during which they remained abstinent for six months or more before relapsing. While this proportion is not unusual in Australian cohorts (Winstock et al., 2011) it may not be representative of people on OAT in different jurisdictions.

Another limitation is whether the study had a long enough follow-up period (16 weeks after last dose). Modelling of plasma buprenorphine concentrations following cessation of Buvidal Monthly suggests that plasma concentrations should fall to negligible levels (below 0.1 ng/ml) about three months (82.3–104.0 days) after the last dose (Björnsson et al., 2023), suggesting our follow up period was sufficient to capture peak withdrawal symptoms.

The small sample size, absence of a comparison group, and inpatient setting prevent us from drawing firm conclusions or recommendations for clinicians. It should be noted for example that risk of overdose increases dramatically after discontinuing OAT, especially in the first four weeks after discontinuation (Jones et al., 2022, Kimber et al., 2015). All clients considering discontinuing OAT should discuss the potential risks and benefits of discontinuing carefully with their clinicians before making a decision. The study recruited a select group of participants without significant medical, psychiatric or substance use comorbidities. The small number of participants prevents us from generalizing to broader populations given the potential for inter-individual variation in buprenorphine metabolism and in the subjective experience of opioid withdrawal. Nevertheless, our findings are consistent with the few small case reports showing mild withdrawal following discontinuation from depot buprenorphine (Ritvo et al., 2021, Sobel et al., 2004, Rodriguez and Suzuki, 2023). We found no evidence of deterioration in physical or mental health scores (PROMIS-29 or DASS-21). Whilst full sleep outcomes (actigraphy and subjective measures) will be reported at a later time, PROMIS-29 subscales suggest no severe disruption to sleep among our participants, nor increase in depression or anxiety symptoms. Although craving scores remained low during the first 6–8 weeks there was a noticeable small average increase in cravings during weeks 8–16 – and not consistent with the timeframe of peak withdrawal severity. This highlights that cravings can occur independently of withdrawal symptoms, and that the risk of resumption of opioid use remains in patients stopping LAIB treatment despite minimal withdrawal discomfort, and stresses the need for ongoing support within a recovery framework for months after discontinuing medication. Successful cessation of OAT requires more than just managing withdrawal symptoms – and patients and service providers should plan for ongoing support, and the option of return to OAT if patients resume regular opioid use.

In conclusion, this case series is consistent with prior case reports and clinical experience that cessation of LAIB treatment is associated with minimal withdrawal discomfort under controlled conditions. Large-scale randomized clinical trials are required to establish whether cessation of LAIB is associated with better clinical outcomes than cessation of sublingual buprenorphine or methadone – which ultimately would guide clinician and patient decision making as to optimal treatment approaches.

## CRediT authorship contribution statement

**SAHID ARSHMAN:** Writing – review & editing, Project administration. **SOMOGYI ANDREW A:** Writing – review & editing, Methodology, Formal analysis, Data curation. **LINTZERIS NICHOLAS:** Writing – review & editing, Writing – original draft, Supervision, Project administration, Methodology, Investigation, Funding acquisition, Conceptualization. **Mills Llewellyn:** Writing – review & editing, Writing – original draft, Visualization, Project administration, Methodology, Investigation, Formal analysis, Data curation. **HAYES VICTORIA:** Writing – review & editing, Writing – original draft, Project administration, Methodology, Investigation. **STUBLEY CAROLYN:** Writing – review & editing, Project administration, Conceptualization. **BYRON GAYE:** Writing – review & editing, Supervision, Project administration. **TREVITT BENJAMIN T:** Writing – review & editing, Project administration. **BLACK ELEANOR:** Writing – review & editing, Project administration.

## Primary funding

South Eastern Sydney Local Health District and Camurus International Pty Ltd.

## Declaration of competing interest

The study was partially funded by Camurus International Pharmaceutical company, manufacturer of Buvidal®. Camurus had no involvement in study planning, conduct, analysis, interpretation or reporting.

## Declaration of Competing Interest

The authors declare the following financial interests/personal relationships which may be considered as potential competing interests:The authors declare financial support was provided by Camurus Pty Ltd - manufacturers of Buvidal -for expenses related to the conduct of this trial, however Camurus had no input into the design, collection, analysis, or interpretation of the data presented in this manuscript. There are no other competing interests to report.
