# Supplementary material for: Characterizing withdrawal from long-acting injectable buprenorphine: An observational case series
Source: Drug Alcohol Depend Rep. 2025 Apr 5;15:100329. doi: 10.1016/j.dadr.2025.100329 (PMC12018199; doi:10.1016/j.dadr.2025.100329)
Supplement: Supplementary file 1 — Supplementary material [file mmc1.docx]

Characterizing withdrawal from long-acting injectable buprenorphine: an observational case series of withdrawal from Buvidal.

**Online Supplementary Material**

Note: The graphs below show COWS, SOWS, OOWS, and Opioid Craving Scale data for people who commenced the study. Two of these chose Sublingual Buprenorphine (eFigures 1 and 3: red lines and points; eFigure 2: dotted lines), and ten did not stay for more than 28 days (eFigures 1 and 3: blue lines and points; eFigure 2: dashed lines) and thus these 12 participants were not analysed for the study. Fifteen participants stayed more than four weeks (eFigures 1and 3: black lines and points; eFigure 2: solid lines). The 27^th^ participant, Participant 19, is one of the 10 participants who commenced the study but who did not stay for more than four weeks. They are one of the three who self-discharged (see ‘Commenced study but did not stay for ≥4 weeks’ box, Figure 1, main manuscript). However, their data is not included in the graphs below as their Day 1 nurse review – containing the COWS, SOWS, OOWS, and Opioid Craving Scale – was misplaced (and hence not entered) and they left unexpectedly and suddenly on Day 9 without any more data being collected. Thus the individual graphs below show only 26 of the 27 participants who commenced the study.


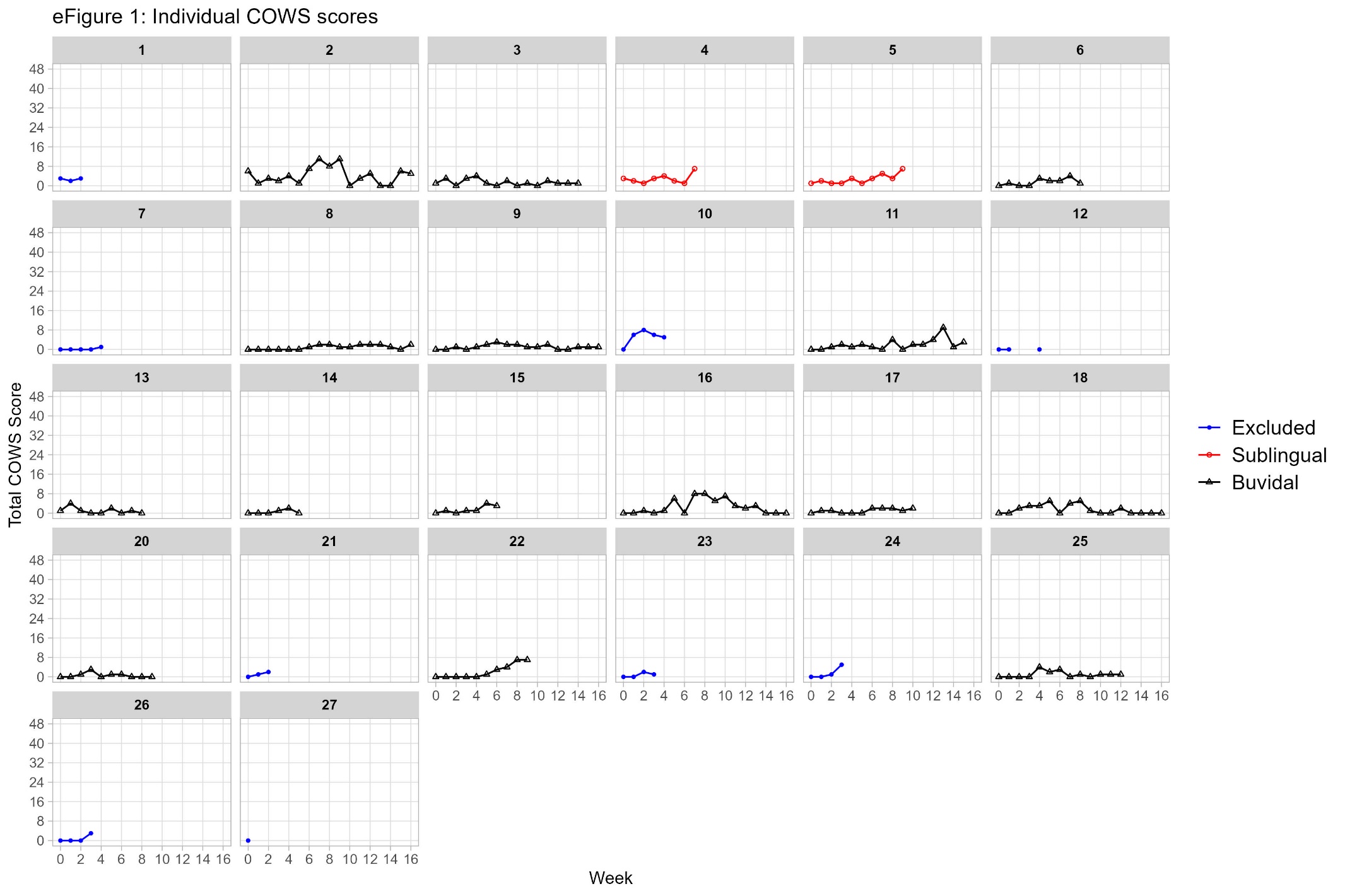


**
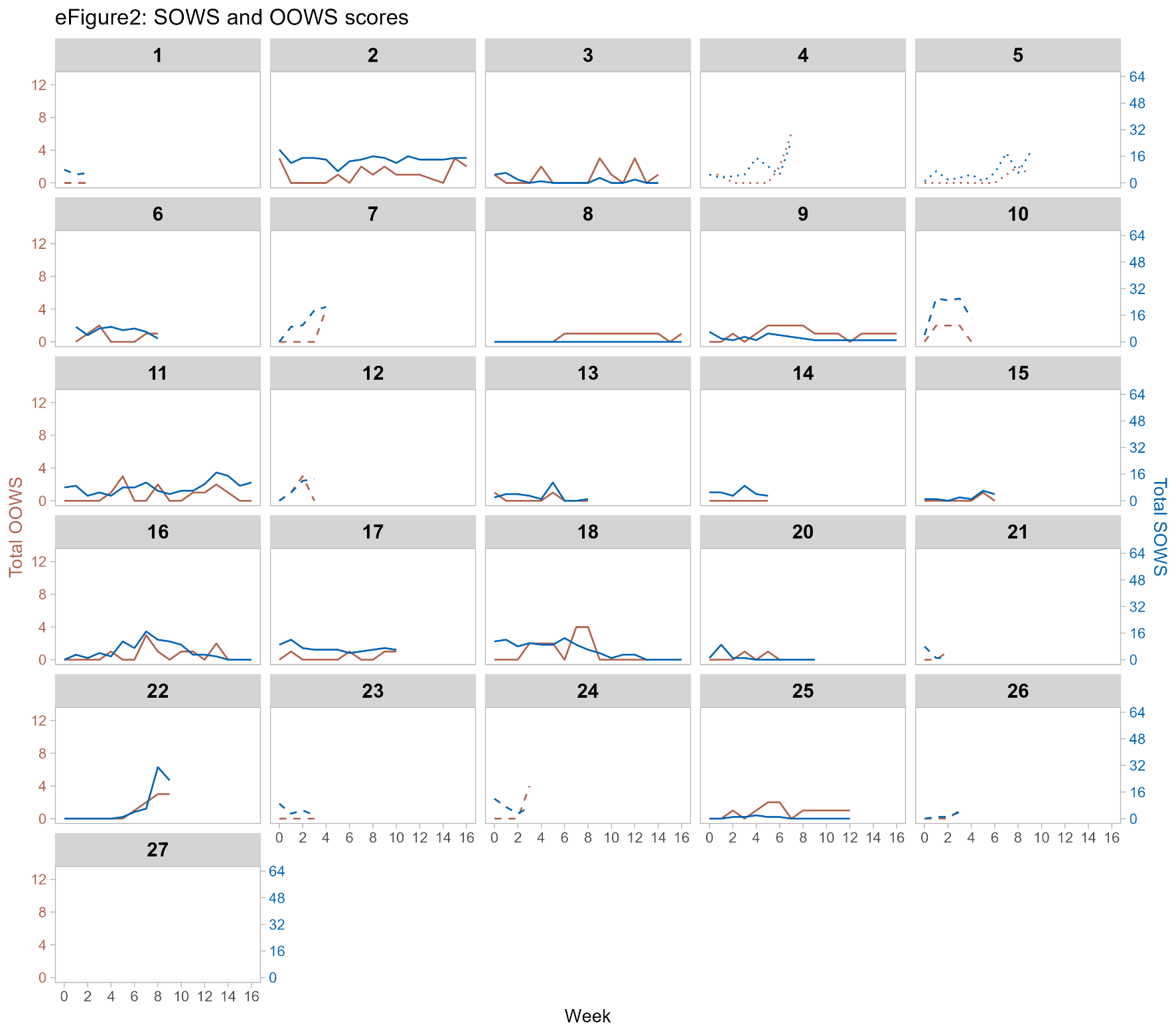
**

= SOWS scores

= OOWS scores

Solid lines = Buvidal

Dotted lines = Sublingual

Dashed lines = Excluded

**
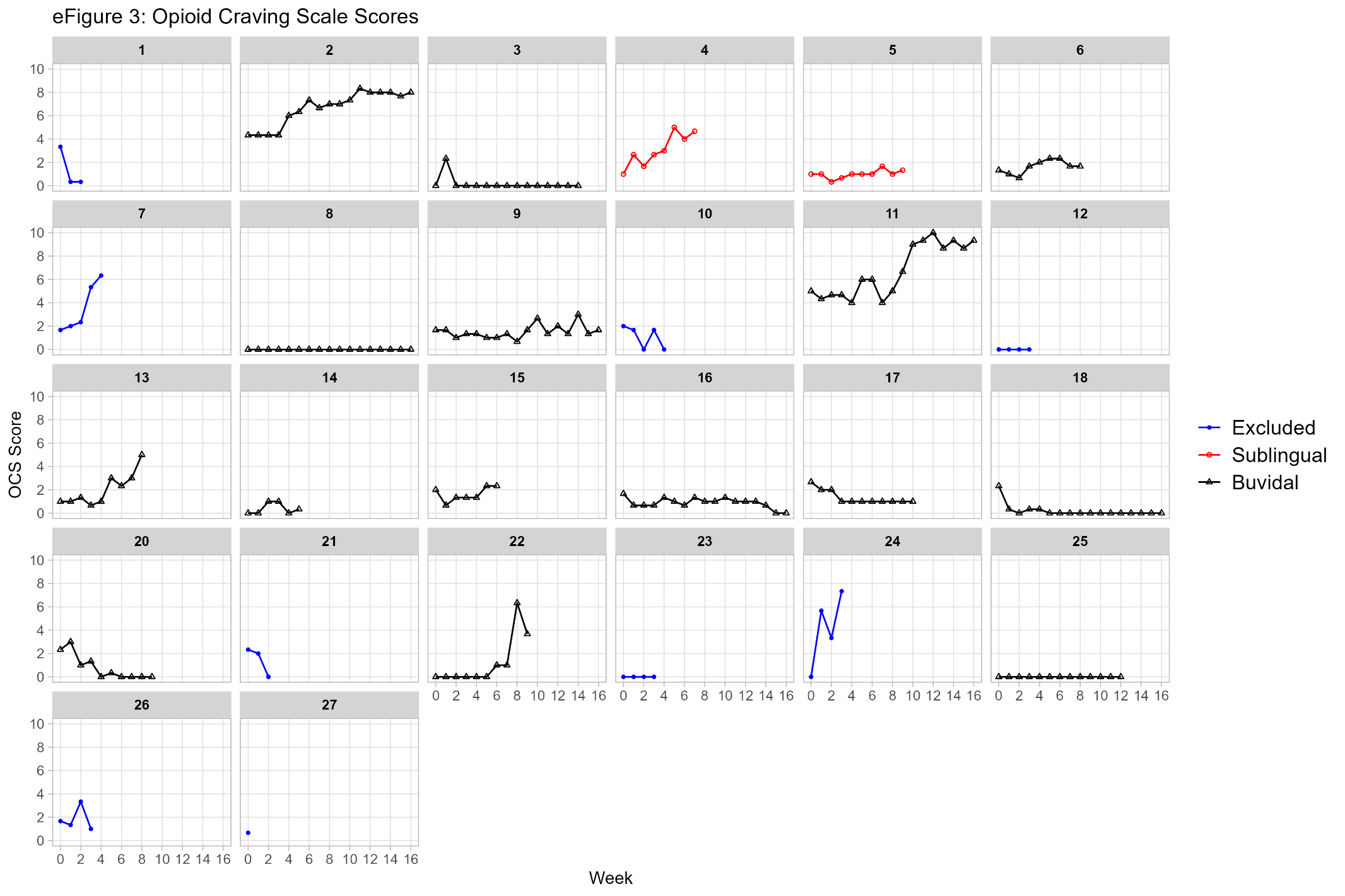
**

**eTable 1.** Rescue medications available as part of the study.

| **Symptom** | **Medication and daily dose** | **Number days / duration** |
| --- | --- | --- |
| Headaches, body aches and pains | Paracetamol 1000mg QID prn  Ibuprofen 400mg TDS prn | 5 days over the admission  5 days over the admission |
| Nausea, vomiting | Metoclopramide 10mg TDS |  |
| Sleep disturbances | Temazepam 10mg nocte | On any 5 nights over the admission but not on consecutive nights. |
| Abdominal cramps | Hyoscine butylromide (Buscopan) | 5 days over the admission |
| Diarrhoea | Lomotil i-ii BD prn | 5 days over the admission |

**Description of dose taper for n=2 participants who chose to taper off sublingual buprenorphine**

Participants in the SL BPN arm underwent a gradual inverse exponential taper over an eight week period based upon the initial SL BPN ‘starting dose’, with reductions of 2-4 mg per week until doses of 8 mg were reached, and then 2 mg per week dose reductions below 8 mg (standard care dose taper for the residential unit).

**eTable 2** Prior distributions for bivariate regression models

| **Outcome/analysis type** | **Regression term** | **Prior Distribution** |
| --- | --- | --- |
| Numeric/Gaussian regression | Intercept  b  sigma | Student’s *t* (3, 0-9.6^a^, 2.5)  Flat  Student’s *t* (3, 0, 2.5) |

**a:** *mean in prior on Intercept varies depending on data, based on an algorithm in the brm() function, but is always broad and weakly regularising to ensure the prior has minimal influence on the posterior*

**eTable 3:** Baseline characteristics for participants included in the study and those excluded for staying les than 29 days and choosing to withdraw from sublingual buprenorphine

|  | **Buvidal in study**  **(*n*=15)** | **Buvidal excluded from study (stayed ≤ 28 days *n*=10)** | **Sublingual Excluded from study (*n*=2)** |
| --- | --- | --- | --- |
| **Age**, yrs-old, M (SD) | 37.1 (5.2) | 36.7 (8.4) | 39.0 (0.0) |
| **Gender**, n (%)  Male  Female | 11 (73%)  4 (27%) | 6 (60%)  4 (40%) | 2 (100%)  0 (0%) |
| **Aboriginal**, n (%) | 3 (20%) | 5 (50%) | 0 (0%) |
| **Education**, n (%)  Left before Yr 10  Attained Yr 10  Attained Yr 12  Attained Tertiary | 4 (27%)  5 (33%)  2 (13%)  4 (27%) | 3 (30%)  3 (30%)  3 (30%)  1 (10%) | 2 (100%)  0 (0%)  0 (0%)  0 (0%) |
| **Living situation**, n (%)  Renting  Own house/mortgage  Lives with family/friends  Homeless | 6 (40%)  1 (7%)  4 (27%)  4 (27%) | 2 (20%)  1 (10%)  5 (50%)  1 (10%) | 1 (50%)  0 (0%)  0 (0%)  1 (50%) |
| **Relationship status**, n (%)  Single/Separated  Married/Defacto | 11 (73%)  4 (27%) | 9 (90%)  1 (10%) | 1 (50%)  1 (50%) |
| **Has children**, n (%) | 6 (40%) | 5 (50%) | 1 (50%) |
| **Current legal issues**, n (%) | 8 (53%) | 5 (50%) | 0 (0%) |
| **Any substance use in previous 28 days**, n (%)  Alcohol  Cannabis  Amphetamine  Benzodiazepine  Heroin | 1 (7%)  0 (0%)  3 (20%)  4 (27%)  2 (13%) | 1 (10%)  1 (7%)  2 (20%)  2 (20%)  1 (10%) | 0 (0%)  0 (0%)  0 (0%)  0 (0%)  0 (0%) |
| **Opioid use history**  Age of first regular use, yrs-old, M (SD)  Age of first opioid treatment, yrs-old, M (SD)  Heroin main type of opioid, n (%)  Came off OAT for 1 month or more once or more, n (%)  When came off OAT, stayed off for ≥6 months, n (%) | 21.7 (6.8)  26.4 (7.4)  14 (93%)  12 (80%)  6/12 (50%) | 19.1 (5.2)  24.9 (6.4)  9 (90%)  8 (80%)  7/8 (88%) | 29.5 (4.9)  31.5 (4.9)  1 (50%)  1 (50%)  1/1 (100%) |
| **Depression Anxiety Stress Scale**, n (%)  Depression  Normal  Mild  Moderate  Severe/Extremely Severe  Anxiety  Normal  Mild  Moderate  Severe/Extremely Severe  Stress  Normal  Mild  Moderate  Severe/Extremely Severe | 8 (53%)  1 (7%)  3 (20%)  3 (20%)  6 (40%)  5 (33%)  3 (20%)  1 (7%)  7 (47%)  3 (20%)  4 (27%)  1 (7%) | 4 (40%)  5 (50%)  1 (10%)  0 (0%)  5 (50%)  3 (30%)  2 (20%)  0 (0%)  6 (60%)  1 (10%)  2 (20%)  1 (10%) | 2 (100%)  0 (0%)  0 (0%)  0 (0%)  1 (50%)  1 (50%)  0 (0%)  0 (0%)  1 (50%)  1 (50%)  0 (0%)  0 (0%) |
| **Detoxification Fear Survey Schedule^a^** – 14, n (%)  Problematic fear of withdrawal  No problematic fear of withdrawal | 5 (33%)  10 (67%) | 6 (60%)  4 (40%) | 1 (50%)  1 (50%) |
| **Treatment Satisfaction Questionnaire – Medication**, M (SD)  Convenience  Effectiveness  Side effects  Global satisfaction | 79.1 (15.4)  75.9 (13.9)  88.3 (17.3)  75.2 (21.2) | 80.6 (14.2)  77.8 (14.6)  90.0 (18.7)  81.4 (14.8) | 72.2 (NA^b^)  72.2 (NA^b^)  100 (NA^b^)  92.9 (NA^b^) |

*Note: Cells may not sum to 100% due to rounding error.* ***a:*** *Problematic fear of withdrawal for DFSS-14: ≥ 34 (Milby et al., 1987).* ***b.*** *One of the two Sublingual participant had no TSQM data collected at baseline.*

**Table 1:** Baseline characteristics (*N*=15)

| **Age**, yrs-old, M (SD) | 37.1 (5.2) | 39.0 (0.0) |
| --- | --- | --- |
| **Gender**, n (%)  Male  Female | 11 (73%)  4 (27%) | 2 (100%)  0 (0%) |
| **Aboriginal**, n (%) | 3 (20%) |  |
| **Education**, n (%)  Left before Yr 10  Attained Yr 10  Attained Yr 12  Attained Tertiary | 4 (27%)  5 (33%)  2 (13%)  4 (27%) |  |
| **Living situation**, n (%)  Renting  Own house/mortgage  Lives with family/friends  Homeless | 6 (40%)  1 (7%)  4 (27%)  4 (27%) |  |
| **Relationship status**, n (%)  Single/Separated  Married/Defacto | 11 (73%)  4 (27%) |  |
| **Has children**, n (%) | 6 (40%) |  |
| **Current legal issues**, n (%) | 8 (53%) |  |
| **Any substance use in previous 28 days**, n (%)  Alcohol  Cannabis  Amphetamine  Benzodiazepine  Heroin | 1 (7%)  0 (0%)  3 (20%)  4 (27%)  2 (13%) |  |
| **Opioid use history**  Age of first regular use, yrs-old, M (SD)  Age of first opioid treatment, yrs-old, M (SD)  Heroin main type of opioid, n (%)  Came off OAT for 1 month or more once or more, n (%)  When came off OAT (*n*=12), stayed off for ≥6 months, n (%) | 21.7 (6.8)  26.4 (7.4)  14 (93%)  12 (80%)  6 (50%) |  |
| **Depression Anxiety Stress Scale**, n (%)  Depression  Normal  Mild  Moderate  Severe/Extremely Severe  Anxiety  Normal  Mild  Moderate  Severe/Extremely Severe  Stress  Normal  Mild  Moderate  Severe/Extremely Severe | 8 (53%)  1 (7%)  3 (20%)  3 (20%)  6 (40%)  5 (33%)  3 (20%)  1 (7%)  7 (47%)  3 (20%)  4 (27%)  1 (7%) |  |
| **Detoxification Fear Survey Schedule^a^** – 14, n (%)  Problematic fear of withdrawal  No problematic fear of withdrawal | 5 (33%)  10 (67%) |  |
| **Treatment Satisfaction Questionnaire – Medication**, M (SD)  Convenience  Effectiveness  Side effects  Global satisfaction | 79.1 (15.4)  75.9 (13.9)  88.3 (17.3)  75.2 (21.2) |  |
